# Improvement of Endothelial Cell-Polycaprolactone Interaction through Surface Modification via Aminolysis, Hydrolysis, and a Combined Approach

**DOI:** 10.1155/2023/5590725

**Published:** 2023-12-13

**Authors:** Femke Bellen, Elisa Carbone, Pieter Baatsen, Elizabeth A. V. Jones, Fatemeh Kabirian, Ruth Heying

**Affiliations:** ^1^Cardiovascular Developmental Biology, Department of Cardiovascular Sciences, KU Leuven, Leuven, Belgium; ^2^Centre for Molecular and Vascular Biology, Department of Cardiovascular Sciences, KU Leuven, Leuven, Belgium; ^3^VIB-KU Leuven Center for Brain & Disease Research, Department of Neurosciences, KU Leuven, Leuven, Belgium; ^4^EM-Platform of VIB Bio Imaging Core at KU Leuven, Leuven, Belgium; ^5^Department of Cardiology, CARIM School for Cardiovascular Diseases, Maastricht University, Maastricht, Netherlands

## Abstract

Polycaprolactone (PCL) is a promising material for the fabrication of alternatives to autologous grafts used in coronary bypass surgery. PCL biodegrades over time, allowing cells to infiltrate the polymeric matrix, replacing the biodegrading graft, and creating a fully functional vessel constituted of autologous tissue. However, the high hydrophobicity of PCL is associated with poor cell affinity. Surface modification of PCL can increase this cell affinity, making PCL an improved scaffold material for acellular vascular grafts. In this study, the surface of PCL films was modified by hydrolysis, aminolysis, and the combination thereof to introduce carboxyl, hydroxyl, and amino groups on the surface. Only the hydrolyzed films exhibited a significant increase in their hydrophilicity, although further testing showed that all aminolysis conditions had amino groups on the surface. Furthermore, *in vitro* experiments with human umbilical endothelial cells (HUVECs) were performed to assess changes in cell affinity for PCL due to the surface treatments. PCL treated with sodium hydroxide (NaOH), a hydrolysis reaction, showed a significant increase in endothelial cell adhesion after 24 hours with a significant increase in cell survival after 72 hours. Thus, NaOH treatment improves the biocompatibility and endothelialization of PCL, creating a competent candidate for artificial, acellular, biodegradable vascular grafts.

## 1. Introduction

Coronary heart disease is the most common type of heart disease and the foremost single cause of mortality and loss of disability adjusted for life years globally [1, 2]. Coronary bypass surgery is one of the most frequent treatments for coronary disease using vascular grafts to replace damaged blood vessels [3, 4]. Ideally, autologous artery segments are used for this surgery as they are both compliant and nonthrombogenic [4, 5]. However, autologous grafts are not always available or suitable to use as grafts due to occlusion or pathological changes, specifically in patients suffering from peripheral vascular disease [5–7]. The large number of patients in need of cardiovascular grafts and the associated requirements thus create a demand for alternative solutions [8].

Current synthetic commercial vascular grafts, especially small-diameter grafts, are associated with a high incidence of failure due to thrombus formation and infection [5, 9, 10]. Vascular graft infections are rare, but they are associated with mortality rates between 26 and 55%, depending on the graft location and surgical procedure [11]. Furthermore, *Staphylococcus aureus* and *Escherichia coli* are collectively responsible for a significant part of all medical device-associated infections [12]. This results in a growing demand to fabricate small-diameter vascular grafts with a diameter under 6 mm with improved biocompatibility and adequate mechanical properties that do not inherit a risk for bacterial infection [9, 13].

Biodegradable materials are preferable because the material degrades over time while cells infiltrate the matrix, producing collagen, elastin, and proteoglycans and replacing the degrading material [8]. Thus, a fully functional vessel is created, constituted of autologous tissue, smooth muscle cells, and endothelial cells [14]. Importantly, the material should degrade over years to ensure a prolonged mechanical support for infiltrating cells while keeping the risk of an inflammatory response low and to avoid rupture and aneurysms of the vessel [8, 14]. Furthermore, the graft should not increase the risk for bacterial attachment to avoid vascular graft infections.

Polycaprolactone (PCL) is a biodegradable and biocompatible polyester used in tissue engineering [15, 16]. It is characterized by a rubbery state at a physiological temperature of 37°C leading to superior mechanical properties over other polyesters [17, 18]. In addition, PCL degrades in the physiological environment *in vivo* and can undergo hydrolytic degradation through bulk or surface degradation mechanisms [15, 19–21]. Another advantage of PCL scaffolds is the long degradation time of more than 18 months *in vivo* due to their hydrophobic nature and high level of crystallinity [17, 19, 22]. However, PCL has a poor cell affinity due to its high hydrophobicity and lack of cell-binding sites [16]. Thus, to use PCL as a scaffold material, its hydrophilicity needs to be increased to allow cellular compatibility with endothelial cells forming a neo-endothelial layer at the surface of the graft.

The cellular compatibility of grafts is primarily determined by the surface properties, including morphology, topography, chemical structure, and functional groups [23, 24]. These surface properties can be changed by multiple methods including treatment with drugs, peptides, growth factors, or adhesive proteins, or chemical reactions such as hydrolysis and aminolysis [15, 25–31]. The advantage of chemical surface modification is the possibility of combining it with other treatments due to its simple and short procedural steps. For PCL, hydrolysis in an alkaline medium results in the formation of carboxylates and hydroxyl groups on the graft surface [32]. Aminolysis is another option to functionalize the graft surface by a fast reaction that creates amide bindings and amino groups on the surface [15, 32].

The aim of the present study was to compare the effect of different surface treatments to increase the endothelialization of PCL. Both hydrolysis and aminolysis, as well as a combination thereof, were used to treat PCL films. The effects of these modifications on the hydrophilicity and surface morphology of the PCL films were investigated and the adhesion and survival of endothelial cells to the PCL films were studied. The bacterial adhesion was examined to ensure that the surface treatment does not lead to an increase in bacterial infectivity. The adequate surface modification would bring PCL a step closer to being a promising biomaterial for vascular prosthesis and other applications.

## 2. Methods

### 2.1. PCL Film Preparation

A PCL solution was made by dissolving PCL (Sigma-Aldrich, 440744) in tetrahydrofuran (Merck KgaA, 107025) (10% w/v). Four ml of this solution were poured in a flat-bottomed glass box of 70 × 105 × 85 mm (Heinz Herenz, A11520). To ensure a homogeneous layer, the glass box was shaken gently while the PCL film dried. The resulting PCL film was cut into disks (*d* = 16 mm) using a circular biopsy punch.

### 2.2. Surface Modification

PCL films were put in glass vials and chemically treated to create 6 conditions:

1. A hydrolysis reaction with 10 M sodium hydroxide (NaOH; Sigma-Aldrich, S8045) in distilled water for 1 h at room temperature (RT)
2. A hydrolysis reaction with a different cation with 10 M potassium hydroxide (KOH; Merck KgaA, 105021) in distilled water for 1 h at RT
3. An aminolysis reaction with 10% hexamethylenediamine (HMD; Sigma-Aldrich, H11696) in 2-propanol for 1 h at 37°C
4. A combination of hydrolysis and aminolysis reactions with 10 M NaOH in distilled water for 1 h at RT, washed twice with distilled water, 10% HMD in 2-propanol for 1 h at 37°C
5. A combination of hydrolysis and aminolysis reactions with 10 M KOH in distilled water for 1 h at RT, washed twice with distilled water, 10% HMD in 2-propanol for 1 h at 37°C
6. Distilled water for 1 h at RT used as negative control

The conditions were selected based on existing literature and adapted, if necessary, such that comparison between the different treatment methods was possible [15, 33–37].

After treatment, the PCL films were washed twice with distilled water and left to air dry overnight before further analysis.

### 2.3. Contact Angle Measurement

To analyze the water contact angle, a Drop and Surface Analyser OCA50 (DataPhysics Instruments GmbH) was used. The PCL film samples were fixed on the support plate using tape. A droplet of water was placed on top of the samples and pictures were taken of the droplet and surface. Image analysis was done using edge detection to identify the liquid/gas interface. The Pendent Drop software was utilized to measure the contact angle from the shape of the drop according to the Young–Laplace equation.

### 2.4. Mass Loss Measurement

To analyze whether the hydrolysis reaction affected the mass of PCL films, the films were weighed before and after treatment using an analytical balance (Kern, ALJ 120-4). The PCL films were dried at 37°C overnight and weighed (W0). They were then processed for surface modification as described above. Afterwards, the films were again dried at 37°C overnight and the weight was measured (W). The mass loss (%) was calculated based on the following equation:

(1) $$\begin{array}{c} \text{Mass loss }\left(\%\right)=\left[\frac{\left(W0–W\right)}{W0}\right]*100. \end{array}$$

### 2.5. Morphological Analysis

To investigate the morphology of treated and untreated PCL, 2 films of each condition were analyzed. Before scanning electron microscopy, the samples were coated with 10 nm chromium. Visualization was performed by a scanning electron microscope (SEM; Zeiss Sigma) at the acceleration voltage of 2 KV, using the Digital Micrograph (GMS 3.0) software.

### 2.6. Ninhydrin Assay

To confirm the effectiveness of the aminolysis reaction, PCL discs were incubated with 100 *μ*l of 1 M ninhydrin (Sigma-Aldrich, N4876) in ethanol in microtubes protected from light. The microtubes were put in a water bath at 70°C for 15 minutes. Next, 500 *μ*l dichloromethane (Janssen Chemica, 11.346.94) was added to each tube to dissolve the films, and 500 *μ*l 2-propanol was added to the solutions. After mixing and vortexing, 200 *μ*l of each solution was put in a 96-well plate. The absorbance was measured at 550 nm using an ELISA microplate reader EL808 (BioTek) with a Gen5 Microplate Reader and the Imager Software version 3.02.

### 2.7. Cell Adhesion and Survival

Human umbilical vein endothelial cells (HUVECs; PromoCell, C-12208) were grown in culture using Endothelial Cell Growth Medium MV2 (EGM-2MV; PromoCell, C-22022). Confluent HUVECs at passage 5 were used for seeding in the experimental set-ups.

**Figure 1:**
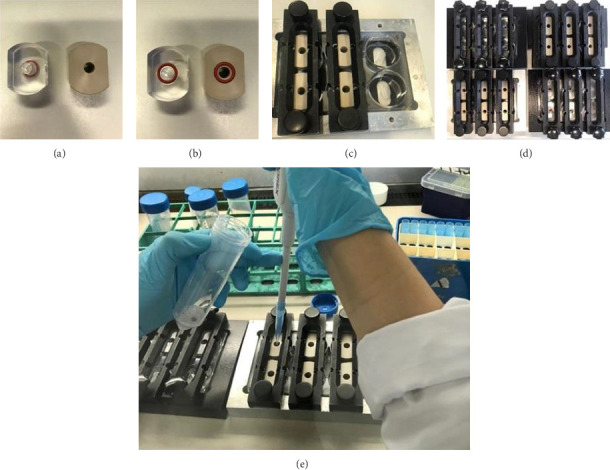
*In vitro* model to seed cells on PCL films. Custom *in vitro* model to seed HUVECs on PCL films using plexiglass inlets, 6-well plates, and metal frames to immobilize PCL films. Plexiglass inlets viewed from the top (a) and bottom (b); metal frame during (c) and after (d) mounting; HUVECs seeding on the surface of sealed PCL films (e).

To seed HUVECs on the PCL films, a custom *in vitro* model was used (designed by Dr. Vogel [38]). Each PCL disk was placed in the center of a well using a 6-well plate. A plexiglass inlet with an opening of 6 mm diameter was put on top of the film, and the system was immobilized by placing the well plate in a metal frame (Figure 1). After immobilization, the setup was UV sterilized for 2 h. HUVECs were seeded at a concentration of 145,000 cells/cm^2^ onto the PCL films and kept at 37°C for 24 or 72 h as noted. The medium was replaced each day. As a positive control, HUVECs were seeded at the same concentration in a 1% gelatin (Sigma-Aldrich, G2500) coated 96-well plate.

### 2.8. Endothelial Cell Staining by DAPI and Phalloidin

After culture, PCL films seeded with HUVECs were washed with Dulbecco's phosphate-buffered saline (DPBS, pH 7.4; Biowest, L0615), fixed in 4% PFA for 15 minutes at RT, then washed 3 additional times with DPBS at 4°C and permeabilized with phosphate-buffered saline with Tween for 20 minutes. The films were blocked with Tris-NaCl-blocking buffer for 1 h at RT and incubated with DAPI (1/1000) and phalloidin staining (1/1000, Abcam, ab176757) for 1 h at RT on a shaker. Afterwards, the films were washed in DPBS and mounted with Prolong Gold (ThermoFisher Scientific, P36930). Images for quantification were taken using an AxioVert 200M microscope (Zeiss). For quantification, 10 images per sample were taken, and the number of cells was calculated using Fiji-imaging software [39]. Images to visualize the cell morphology were taken using an Axio Imager Z1 (Zeiss).

### 2.9. Bacterial Adhesion

The adhesion of *S. aureus* ATCC 8325-4 and *E. coli* 25922 to treated PCL films was investigated. Bacteria were stored in liquid medium supplemented with 15% (vol/vol) glycerol (ThermoFisher Scientific, 17904) at −80°C. Prior to the experiment, single colonies of the respective bacteria were picked up for overnight culture. *S. aureus* ATCC 8325-4 was grown in 10 ml Tryptic Soy Broth (Sigma-Aldrich, 22092) and *E. coli* ATCC 25922 in 10 ml Luria-Bertani Broth (Sigma-Aldrich, L2542) overnight at 37°C. After two washing steps with DPBS, the concentration was assessed spectrophotometrically at 600 nm (UV-vis) using a BioPhotometer (Eppendorf). PCL films were incubated in 2 ml of 1 × 10^7^ bacteria/ml *S. aureus* and *E. coli* suspension for 1 h under shaking at 200 rpm at 37°C. After being washed twice, the films were transferred to 1 ml of PBS and the adhered bacteria were detached from the films by using the ultrasound cleaner (VWR) for 20 minutes. Serial dilutions were made from the initial inoculation dose and each solution containing the detached bacteria and the respective PCL samples. Bacterial solutions were plated on an agar plate and incubated overnight at 37°C to count colony-forming units (CFUs). Results are expressed as a percentage of adhesion of the total amount of bacteria incubated to the respective sample.

### 2.10. Statistical Analysis

Results are presented as mean ± standard error of the mean (SEM). Each data point represents the average value of one film. Statistical analysis was performed using GraphPad 9 Prism. Variance was tested with the *F*-test (*p* < 0.05) and normality was tested with the Shapiro–Wilk test (*p* < 0.05). Biological outliers were picked up based on Grubb's test (*p* < 0.05). The data were analyzed using a one-way ANOVA with Dunnett's *post hoc* test or Tukey's *post hoc* test. If the data were not normally distributed, the Kruskal–Wallis test with Dunn's *post hoc* test was used.

## 3. Results

### 3.1. Surface Characterization of Treated PCL

#### 3.1.1. SEM Analysis

**Figure 2:**
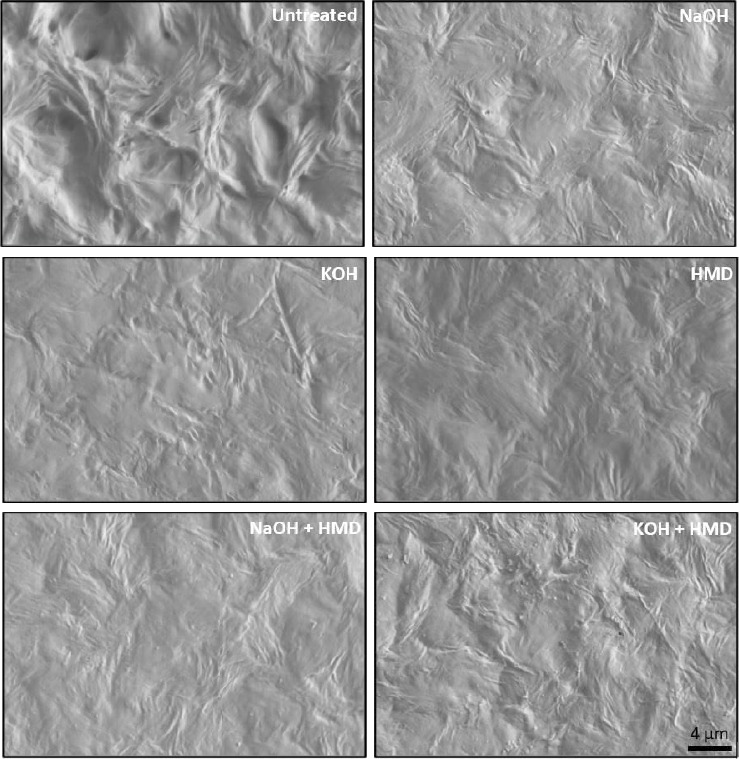
Modified surface of treated PCL samples. SEM images of untreated PCL and films treated with NaOH, KOH, HMD, NaOH + HMD, and KOH + HMD, *n* = 2. Representative images of each condition. Scale bar: 4 *μ*m.

The surface modification of the PCL films was confirmed by morphology analysis using SEM. The PCL surface changed remarkably in all conditions (Figure 2). However, there was no rupture of the PCL films after treatment, and the surface was homogenously affected.

#### 3.1.2. Evaluation of Hydrophilicity

**Figure 3:**
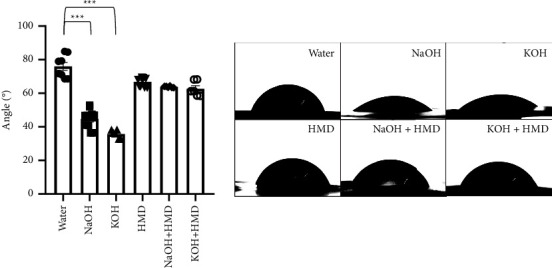
Hydrophilicity of treated PCL films. Contact angle measurements of PCL films treated with water, NaOH, KOH, HMD, NaOH + HMD, and KOH + HMD, *n* = 6 – 10. Data are presented as mean ± SEM. Statistical significance was tested with Kruskal–Wallis test. ⁣^*∗∗∗*^*p* < 0.001.

To study the effect of the surface treatments on the hydrophilicity of the PCL, the contact angles were measured. The wettability was significantly increased on PCL films modified by hydrolysis compared to control PCL films treated with water (Figure 3). There was a significant increase in hydrophilicity on the films modified with KOH (35.638° ± 1.012°, *p* = 0.001) and NaOH (44.724° ± 1.843°, *p* = 0.001). However, there was no significant difference when comparing the two hydrolysis treatments. The combined treatments of NaOH + HMD (63.759° ± 0.180°) and KOH + HMD (62.628° ± 1.820°) as well as the HMD treatment alone (66.783° ± 0.847°) showed no significant decrease in the contact angle compared to PCL films treated with water (75.923° ± 2.407°). In addition, there was no significant difference in the contact angle between films treated with HMD and films treated with HMD in combination with NaOH or KOH. Thus, the results showed that hydrolysis treatments, here demonstrated by KOH and NaOH, were more efficient in increasing the hydrophilicity compared to treatment by aminolysis or the combination of aminolysis and hydrolysis.

#### 3.1.3. Mass Loss

**Figure 4:**
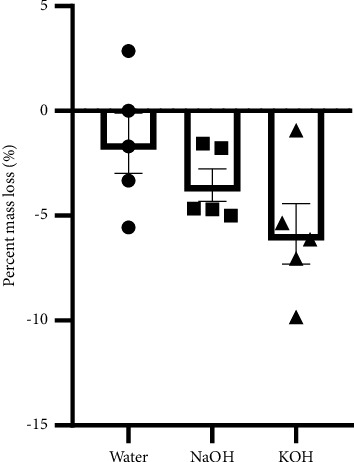
Loss in mass of PCL films modified by hydrolysis and water. Weighing of PCL films treated with water, NaOH, and KOH, *n* = 5. Data are presented as mean ± SEM. Statistical significance was tested with Kruskal–Wallis test.

To study whether the hydrolysis surface modification led to any mass loss, the weight of PCL films was monitored before and after the surface modification process (Figure 4). A slight loss in mass was observed in both treatment conditions (NaOH −3.5% ± 0.8%; KOH −5.9% ± 1.4%). However, these changes were not significantly different from the water-treated control films (−1.5% ± 1.4%).

#### 3.1.4. Ninhydrin Assay

**Figure 5:**
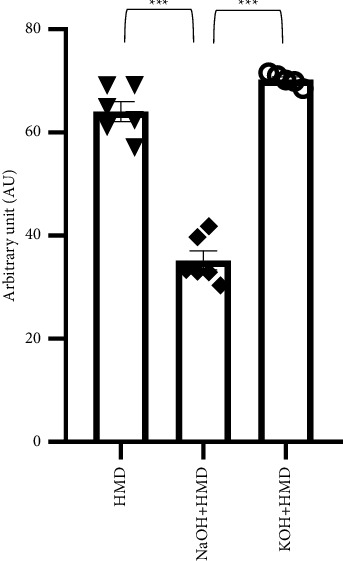
Ninhydrin assay of PCL modified by aminolysis. Ninhydrin assay of PCL films treated with HMD, NaOH + HMD, and KOH + HMD, *n* = 5-6. Data are presented as mean ± SEM. Statistical significance was tested with one-way ANOVA. ⁣^*∗∗∗*^*p* < 0.001.

To exclude the possibility that aminolysis failed to affect the wettability, alone or in combination with hydrolysis, we performed a ninhydrin assay on the PCL films to confirm the formation of amide bindings. Amino groups were present on all treated conditions, confirming the efficiency in the formation of amide bindings (Figure 5). A significantly higher number of amino groups was detected on PCL films treated with KOH + HMD (70.232 ± 0.521, *p* = 0.001) and with HMD (64.033 ± 1.931, *p* = 0.001) alone than on PCL films treated with the combination of NaOH and HMD (35.184 ± 1.835).

### 3.2. Endothelialization

**Figure 6:**
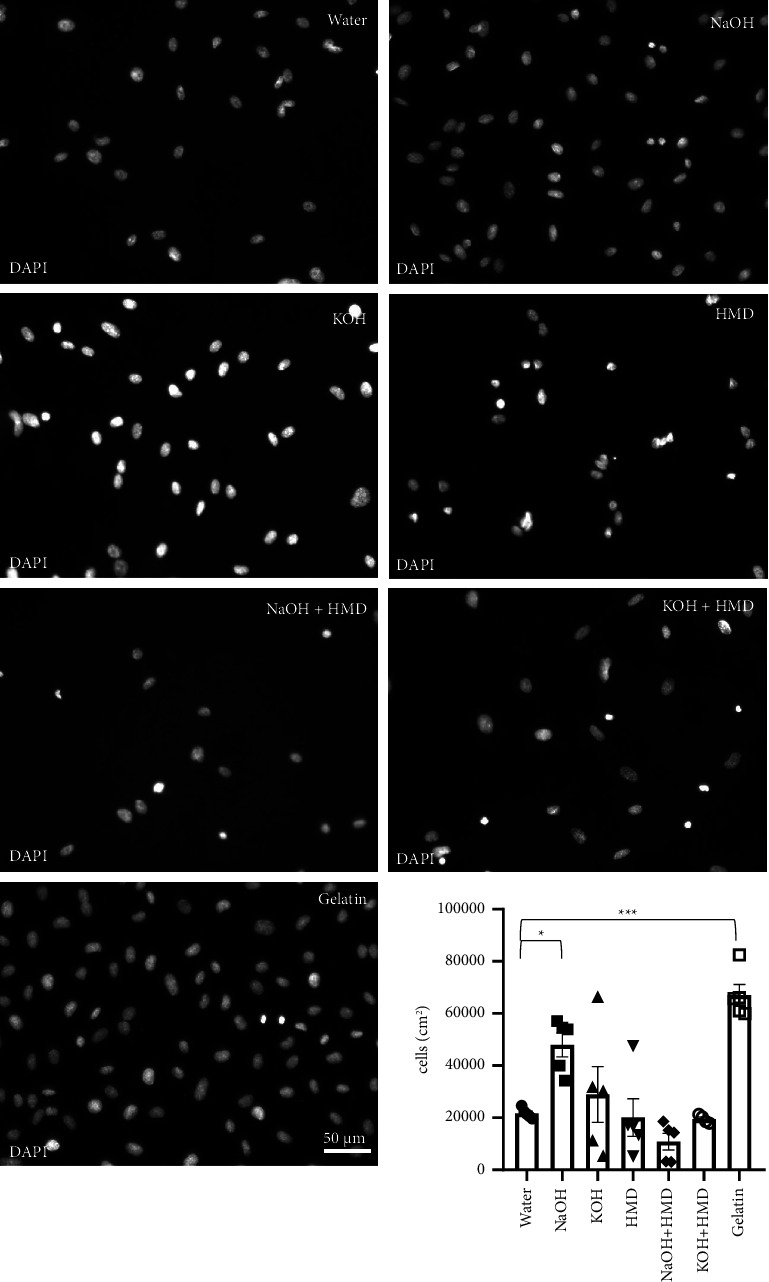
Cell adhesion on PCL films after 24 h. DAPI staining and quantification of HUVECs seeded on gelatin and on PCL films treated with water, NaOH, KOH, HMD, NaOH + HMD, and KOH + HMD. The cells were left to grow for 24 h *n* = 4-5. Representative images of each condition. Data are presented as mean ± SEM. Statistical significance was tested with one-way ANOVA. ⁣^*∗*^*p* < 0.05, ⁣^*∗∗∗*^*p* < 0.001. Scale bar: 50 *μ*m.

To study the effect of surface treatments on cell adhesion to PCL, HUVECs were seeded on chemically treated PCL films. Analysis of the DAPI staining showed that there was a significant increase in cell adhesion to films treated with NaOH (48000 ± 4600 cells/cm^2^, *p* = 0.014) after 24 h (Figure 6). All other treatments displayed no significant difference with the control condition, where PCL was treated with water (22000 ± 900 cells/cm^2^). HUVECs seeded on 1% gelatin were used as a positive control (67000 ± 4000 cells/cm^2^, *p* = 0.001).

**Figure 7:**
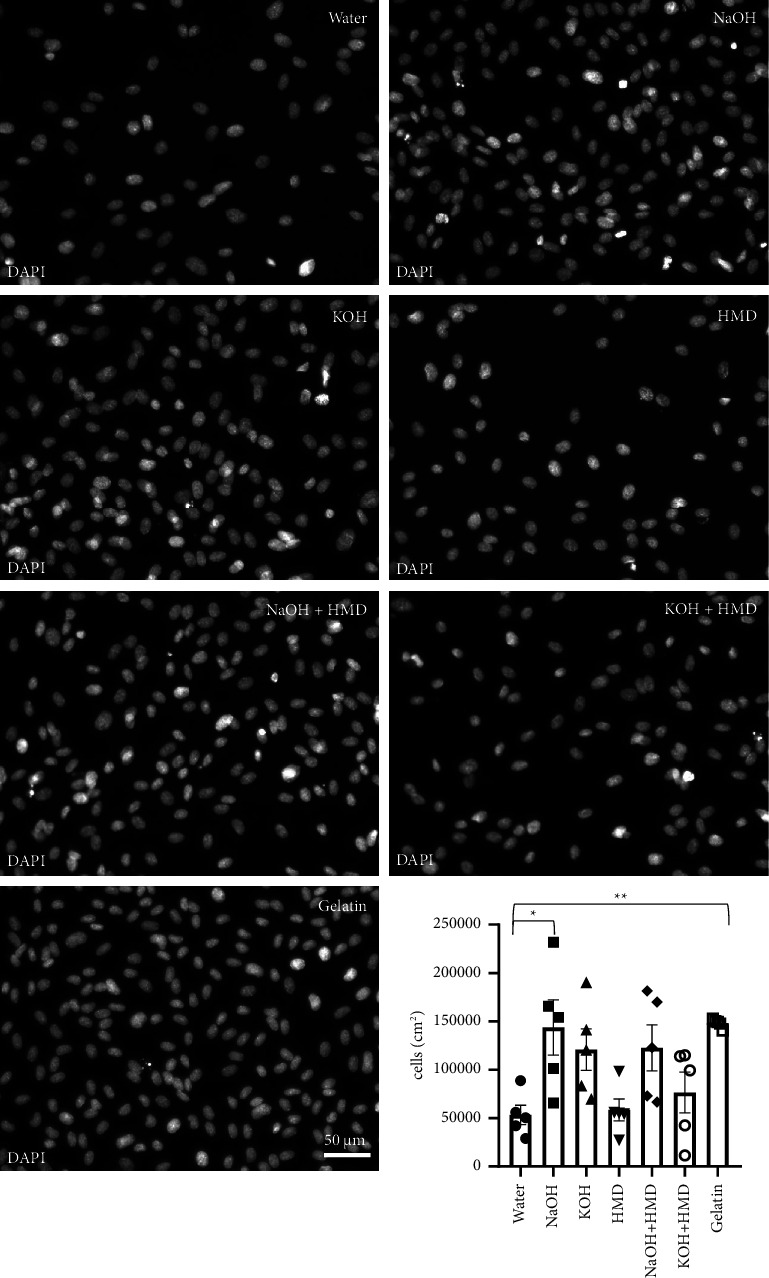
Cell survival on PCL films after 72 h. DAPI staining and quantification of HUVECs seeded on gelatin and on PCL films treated with water, NaOH, KOH, HMD, NaOH + HMD, and KOH + HMD. The cells were cultured for 72 h *n* = 5. Representative images of each condition. Data are presented as mean ± SEM. Statistical significance was tested with one-way ANOVA. ⁣^*∗*^*p* < 0.05, ⁣^*∗∗*^*p* < 0.01. Scale bar: 50 *μ*m.

After 72 h, PCL films treated with NaOH (144000 ± 28500 cells/cm^2^, *p* = 0.011) were still the only condition that had a significantly higher number of cells than films treated with water (53000 ± 10000 cells/cm^2^) (Figure 7). HUVECs seeded on 1% gelatin were used as a positive control (148000 ± 2100 cells/cm^2^, *p* = 0.007), and the number of cells on NaOH-treated PCL approached the levels of this positive control. In addition, films treated with KOH (121000 ± 21500 cells/cm^2^, *p* = 0.079) and NaOH + HMD (123000 ± 23800 cells/cm^2^, *p* = 0.068) had higher cell counts than the water-treated films, although these were not significant because of the variability. PCL modified by HMD (58000 ± 11400 cells/cm^2^) and KOH + HMD (76000 ± 21000 cells/cm^2^) showed no difference with the negative control.

**Figure 8:**
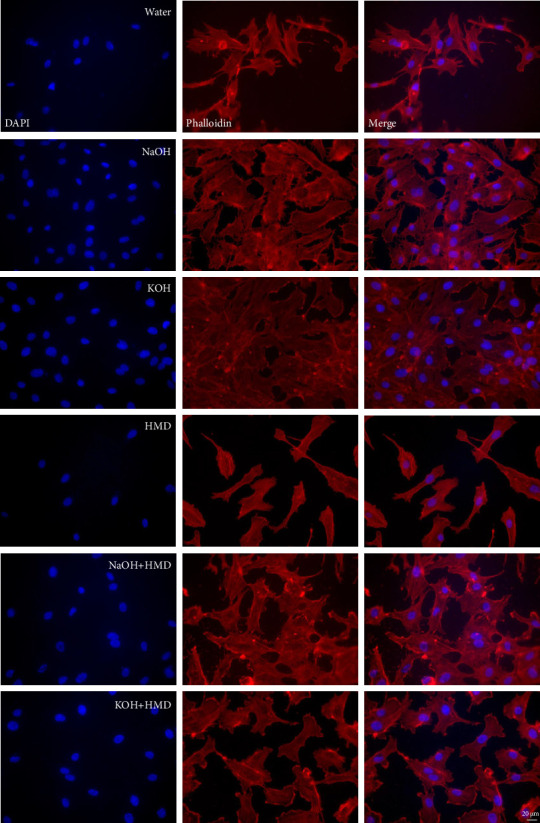
Structure of cells grown on PCL films after 72 h. Phalloidin staining of HUVECs seeded on PCL films treated with water, NaOH, KOH, HMD, NaOH + HMD, and KOH + HMD. The cells were cultured for 72 h *n* = 2. Representative images of each condition. Scale bar: 20 *μ*m.

A phalloidin staining showed that HUVECs grown on PCL films had a normal cytoskeleton structure and cell morphology after 72 h. There was no difference in cytoskeleton structure and thus cell morphology between HUVECs seeded on water-treated PCL films and on films with a surface treatment (Figure 8).

### 3.3. Bacterial Adhesion

**Figure 9:**
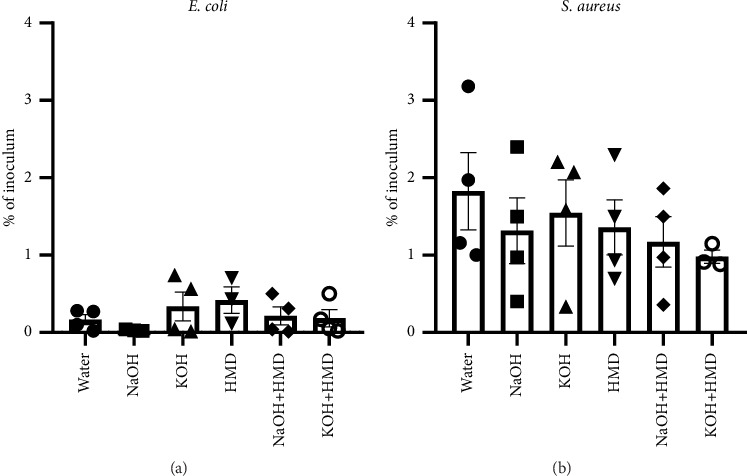
Bacterial adhesion to PCL films. (a) PCL films incubated in *E. coli* 1 × 10^7^ CFU/ml for 1 h, *n* = 3-4. (b) PCL films incubated in *S. aureus* 1 × 10^7^ CFU/ml for 1 h, *n* = 3-4. Data are presented as mean ± SEM. Statistical significance was tested with one-way ANOVA.

To investigate the effect of chemical surface treatments on bacterial adhesion, treated PCL films were incubated with *S. aureus* and *E. coli*. CFU counting after bacterial detachment revealed that *S. aureus* inherently shows a higher adhesion to PCL compared to *E. coli* (Figure 9). However, both tested bacteria displayed no significant difference in adhesion for surface treatment conditions compared to water-treated films.

## 4. Discussion

As autologous grafts are often unavailable for coronary artery bypass surgery, there is a high demand for alternative solutions such as biocompatible, artificial grafts [3, 5]. PCL, being a biodegradable polyester, is a possible candidate for the fabrication of cardiovascular grafts after optimizing the cell compatibility and ensuring no increase in bacterial infectivity. To increase the endothelial cell affinity, the surface of PCL films was modified by hydrolysis, aminolysis, or a combination of both. Current results show that NaOH treatment leads to the best surface modification to increase endothelial cell adhesion and survival on PCL films, which cannot be further increased by dual treatment.

Research on the effect of surface treatment of PCL with NaOH has been done in tissue engineering approaches in multiple fields including cardiovascular research, orthopedic research, and neurology as PCL can also be used to make bone grafts or artificial conduits for nerve repair [15, 40, 41]. In our approach, NaOH and KOH were used to introduce -COOH groups on the polymer surface by hydrolysis and -NH_2_ groups via aminolysis by HMD treatment. The effect of the combination of both hydrolysis conditions with aminolysis was also studied, as we wanted to investigate the combined effect of -COOH and -NH_2_ groups.

All conditions were found to affect the surface of PCL. SEM analysis showed an intact surface without signs of degradation, while clear modifications were visible. Surface modification with NaOH is known to increase the groves on the surface, creating more space for cells to adhere [40]. The optimal water contact angle for maximum cell adhesion has been found to be in the region of 45–70°C or in the range of 30–60°C [17]. Contact angles that are too low lead to an increase in water uptake, which decreases the protein adsorption needed for cell attachment [17]. On the other hand, a very high contact angle causes low cell-conductive behavior and protein denaturation [42]. Our contact angle measurements are in the optimal range for all conditions, except the negative control. In addition, the measurements showed that the hydrolysis reactions significantly increased the hydrophilicity, but aminolysis reactions did not. Importantly, hydrolysis and aminolysis have different kinetics [15, 43, 44]. In brief, NaOH and KOH drive the hydrolysis of a surface by nucleophilic attacks of hydroxide ions on the carbonyl carbon, generating carboxylic acid groups, while the aminolysis reaction causes bulk degradation [44]. Interestingly, the combination of hydrolysis and aminolysis showed no difference in contact angle when compared with aminolysis alone. The weight monitoring of PCL films revealed that there was no significant mass loss with either NaOH or KOH treatment. Bosworth et al. previously reported that a 10 M NaOH treatment for 24 h caused between 32% and 52% mass loss [34]. We allowed the hydrolysis reaction to proceed for only 1h, which was likely short enough to not cause any significant mass loss. Thus, the degradation caused by a short 1 hour treatment is negligible while still increasing the hydrophilicity of the PCL films. Furthermore, the ninhydrin assay revealed significantly less amino groups on the surface of NaOH + HMD-treated PCL than on PCL treated with HMD + KOH or HMD alone. One explanation is that the Na + cation is more Lewis acidic than the K+ cation. Lewis acids can activate a substrate towards nucleophilic attacks, which is how the hydrolysis reaction on PCL works [45]. Thus, NaOH could be generating a bigger surface modification than KOH, creating more carboxylic acid groups whereon the aminolysis reaction cannot occur as easily at the temperatures used [46]. The extent of the difference observed in the ninhydrin assay is hard to reconcile with an effect purely due to Lewis acidity. Alternatively, it is possible that on a molecular level, more water is left on the NaOH-treated films, resulting in more hydrolysis.

The endothelialization of biodegradable vascular grafts is needed to ensure that a fully functional vessel can be created, out of autologous tissue, before the degradation of PCL is completed. The current study confirms that NaOH treatment is the most effective surface modification to increase vascular endothelial cell adhesion to PCL films. Unexpectedly, NaOH treatment and KOH treatment showed differentially effects, with NaOH allowing for faster coverage with endothelial cells. After 24 h, NaOH was the only treatment which gave a significant increase in endothelial cell adhesion when compared to PCL films treated with water. This agrees with the research performed by Serrano et al., who demonstrated that NaOH surface treatment increased the cell adhesion of endothelial cells to PCL after 24 h [41]. In addition, Zhu et al. determined that aminolysis, investigated by measuring the adhesion of endothelial cells to PCL treated with 1,6-hexanediamine/2-propanol, does not increase the cell attachment ratio over untreated PCL but improves the cell morphology [33]. We therefore investigated whether the combination of hydrolysis and aminolysis gave improved results. However, we found that NaOH without aminolysis increased the cell adhesion the most. In addition, after 72 h, NaOH was also the only treatment which gave a significant increase in endothelial cell survival. However, there is also a clear increase, although not significant, in cell survival on PCL films treated with KOH and HMD + NaOH. If the effect of KOH on hydrolysis is indeed less pronounced, then longer times may be needed to see an effect on the endothelialization of KOH treated PCL films. The same principle can be applied on PCL modified with HMD + NaOH, suggesting that the effect of NaOH is diluted by the HMD treatment. None of our measurements of SEM and hydrophilicity, however, indicated a difference between NaOH and KOH. The endothelial cell layer was less confluent on the PCL films treated with HMD and KOH + HMD after 72 h but the endothelial cells were growing on all treated surfaces, suggesting that endothelial cells follow their native growth pattern when seeded on PCL films independent of which surface modification is performed.

Tissue engineering attempts to promote ideal conditions for cell adhesion to surfaces have to pay attention to the specificities of various cell types and the required sites for cell attachment. As such, it was important to look specifically at endothelial cell adhesion. Previous studies have demonstrated that NaOH treatment increases the cell adhesion to PCL of different cell types, such as Schwann cells, smooth muscle cells, endothelial cells, and osteosarcoma cells [15, 40, 41]. Contrasting findings reveal that the potential for cell adhesion differs depending on the cell type. de Luca et al. described that HMD, KOH, and NaOH all increased the cell adhesion of Schwann cells to PCL to the same level [15]. On the other hand, Moura et al. found that NaOH does not increase the cell adhesion of bone marrow mesenchymal stem/stromal cells to PCL [47]. In other settings, aminolysis treatment has been proven to be favorable. Lee et al. reported that NH_2_ groups were the most effective at increasing cell adhesion of K100 erythroleukemia cells to self-assembled monolayers of alkylsilanes. They attributed this to the interaction of NH^+^_3_ and negatively charged glycosaminoglycans of the cell membrane [43]. However, different cell types have different requirements for their ideal matrix. Each cell type reacts differently to different surface characteristics and to different polymers. This highlights the value of our study to investigate cell adhesion specifically for cardiovascular application.

There was no significant difference between the negative control and the treated PCL films when looking at bacterial adhesion. These outcomes suggest that the tested treatments do not attract bacterial attachment and thereby do not increase the risk for infection after surface treatment. A higher adhesion of *S. aureus* compared to *E. coli* could be explained by the multiple surface molecules of *S. aureus*, also known as MSCRAMM, which mediate binding to different biological components such as fibrinogen and collagen. Albeit these results show that the surface modification of PCL does not increase the bacterial infectivity, extra antibacterial factors can be added to the vascular graft to ensure an antiinfectious effect of the material. This can be done by adding drug releasing systems, for which PCL is an excellent candidate due to its high drug permeability. There has been promising research conducted on combining drug releasing systems with PCL using antibiotics and nitric oxide as well as other compounds [32, 48–52]. The combination of drug releasing systems or nitric oxide with the NaOH surface modification can possibly increase the endothelization of the graft in a next step while, in addition, decreasing the bacterial infectivity. Thereby, the complications after vascular graft implantation could be decreased, also reducing the mortality rate in patients.

Our research strengthens the preference for NaOH to modify the surface of PCL while simultaneously showing that other hydrolysis reactions, aminolysis reactions, and the combination thereof are less appropriate candidates to increase the endothelialization of PCL used for cardiovascular approaches. While there are other ways of increasing the endothelialization of vascular grafts, such as modifying the polymer graft with zwitterionic polymers or silk fibroin or coating with gelatin, the relative simple method of surface modification tested in this paper opens the possibility of combining the surface modification with other treatments [29, 53, 54]. These drug or gene treatments can then further improve the endothelialization or decrease the bacterial infectivity and platelet activation. Chemical modifications are also possibly stable for longer times. For example, gelatin coating degrades within 14 days in the body, while the re-endothelization takes much longer [55, 56]. In addition, the simple nature of the treatment by NaOH might allow a quicker path through the regulatory approval process, allow longer shelf life, or, at least, result in shorter production times than other possible surface treatments. Hence, NaOH treatment shows great potential as a catalysator of endothelial cell adhesion and survival on PCL. Further research is needed to test NaOH-surface-modified grafts under shear stress, both *in vitro* and later *in vivo.* In a next step, the association of a drug delivery system to the graft can also be explored. Thus, NaOH treatment causes promising strides in the optimalization of PCL as a biodegradable graft material for cardiovascular and other applications.

## Data Availability

The datasets generated and/or analyzed in the current study are available from the corresponding author upon reasonable request.
